# Effects of Soft Ground on Paw Center of Pressure Metrics in Dogs During Walk and Trot

**DOI:** 10.3390/ani16030397

**Published:** 2026-01-27

**Authors:** Christiane Lutonsky, Julia Kohlmann, Bianca Reicher, Kathleen Wittek, Isabella Brauner, Alexander Tichy, Marion Mucha

**Affiliations:** 1 Section of Physical Therapy and Rehabilitation, Small Animals Surgery, Clinical Centre for Small Animal Health and Research, Clinical Department for Small Animals and Horses, University of Veterinary Medicine, 1210 Vienna, Austriamarion.mucha@vetmeduni.ac.at (M.M.); 2 Platform for Bioinformatics and Biostatistics, Department of Biological Sciences and Pathobiology, Centre of Biological Sciences, University of Veterinary Medicine, 1210 Vienna, Austria

**Keywords:** pressure plate measurement, paw center of pressure, vertical ground reaction forces, canine rehabilitation, physical therapy, postural stability

## Abstract

Physiotherapeutic interventions in dogs frequently incorporate compliant surfaces to improve postural stability (PS), proprioception, and limb coordination; however, objective assessment of surface-related locomotor adaptations remains limited. This study investigated the effects of walking on surfaces with increasing compliance on PS in healthy adult dogs using paw center of pressure (pCOP) analysis. Fourteen orthopedically and neurologically sound dogs were assessed at walk and trot on a pressure-sensitive walkway under four surface conditions: a standard rubber mat (0.1 cm) and three yoga mats of increasing thickness (0.5 cm, 0.8 cm, and 1.0 cm). Vertical ground reaction force (vGRF) and pCOP parameters were recorded and analyzed for each limb. vGRF parameters were largely unaffected by surface conditions. In contrast, several pCOP metrics, including pCOP radius, craniocaudal displacement, and mediolateral displacement, showed a significant decrease with increasing surface compliance. These effects were more consistent and widespread during trot, whereas during walk changes were primarily observed in the forelimbs. The findings indicate that dogs adapt to increased surface compliance by reducing pCOP displacement, likely reflecting active postural stabilization strategies. While vGRF parameters appeared insensitive to these adaptations, pCOP measures proved more sensitive in detecting subtle neuromuscular adjustments. These results highlight the potential value of pCOP analysis for evaluating functional adaptations during physiotherapeutic interventions in canine rehabilitation.

## Simple Summary

Physiotherapy in dogs often includes exercises on soft or unstable surfaces to improve balance, body awareness, and coordinated movement. Despite their frequent use, little is known about how dogs adjust their posture when moving on surfaces of different softness. This study investigated how healthy adult dogs adapt their walking and trotting patterns when moving across surfaces with increasing compliance. Fourteen dogs walked and trotted over a pressure-sensitive walkway covered with mats of different thicknesses. This method allowed the assessment of how body weight was distributed and how the center of pressure beneath each paw moved during ground contact. The overall forces applied to the ground remained largely unchanged across surfaces. However, the movement of the pressure point within the paw decreased on softer surfaces, particularly during trotting. This indicates that dogs actively adjust their posture to maintain stability when moving on compliant ground. These adaptations were more pronounced at faster speeds and were mainly observed in the front limbs during walking. The findings demonstrate that measuring pressure distribution within the paw is a sensitive way to detect subtle balance adjustments. This knowledge may support veterinarians and animal physiotherapists in objectively evaluating and optimizing rehabilitation exercises for dogs.

## 1. Introduction

Maintaining health and mobility in dogs—whether working animals or companion pets—has gained increasing attention in recent decades [1,2,3]. This growing focus includes the development and implementation of physiotherapeutic programs aimed at enhancing musculoskeletal function and overall quality of life. These programs typically target core stability, strength, flexibility, and proprioception [4,5]. Active movement therapy in dogs may include exercises such as gait transitions, uphill or backward walking, obstacle courses, hydrotherapy, and walking or standing on variable surfaces, including soft mats. The goals of these exercises are to improve muscular elasticity and contractility, enhance proprioceptive input, increase soft tissue perfusion, promote balance and coordination, support joint and bone health, and maintain or restore functional movement patterns [4,6].

In veterinary medicine, the use of pressure plates to measure vertical ground reaction forces (vGRFs) has become increasingly prevalent over recent decades, offering an objective and noninvasive method to assess limb-ground interactions during the stance phase. VGRF analysis has been used to characterize normal gait patterns in healthy animals [7,8,9], as an objective diagnostic tool for lameness [10,11,12], and to evaluate the effects of various treatments [13,14]. Commonly derived parameters include peak vertical force (PFz), which represents the maximum vertical force exerted; vertical impulse (IFz), which reflects the total load over time; and time to PFz (TPFz), indicating the timing of peak force within the stance phase [15,16,17].

In contrast to the well-established use of kinematics and electromyography in evaluating physiotherapeutic exercises in dogs [18,19,20,21,22,23,24,25], only a limited number of studies have examined their effects on vGRF and paw center of pressure (pCOP) [16,17]. PCOP analysis offers additional insight into postural control and limb loading. Parameters such as craniocaudal and mediolateral excursion, pCOP speed, area, and radius can be assessed noninvasively using force or pressure measurement systems [12,26]. These objective methods are widely used in both dynamic (e.g., walking, trotting) [12,16,17,26,27,28] and static (e.g., standing) conditions [29,30,31,32,33,34,35]. The pCOP represents the point of application of the resultant vertical ground reaction force vector, which continuously shifts during ground contact to form the so-called pCOP path—a valuable indicator of balance and postural stability [36].

Previous studies using pressure-sensitive plates have investigated the biomechanical effects of walking over obstacles. Significant changes in vGRF and pCOP parameters were observed, particularly in the hindlimbs. Impulse and stance duration increased, while pCOP path length decreased, with the most pronounced changes occurring in the leading hindlimb after crossing one or two obstacles. In contrast, the forelimbs showed minimal alterations [16]. Similarly, wearing dog boots—especially on a single limb—resulted in asymmetrical vGRF distribution and significant changes in pCOP area, particularly in the contralateral limbs [26]. These findings suggest that even moderate external challenges, such as surface irregularities or boot usage, can influence postural control and load distribution in healthy dogs.

Walking on different surfaces significantly affects gait mechanics in humans, as reflected by changes in vGRF and COP parameters. Specifically, surface-dependent adaptations include alterations in the magnitude and timing of the first and second vGRF peaks, changes in loading and unloading slopes, and modifications of COP trajectory characteristics. In addition, walking on compliant or uneven surfaces is associated with increased variability of these parameters. These changes are commonly interpreted as adaptive strategies to altered surface stiffness and stability, allowing individuals to modulate limb stiffness and impact attenuation to maintain overall gait stability and consistent center of mass control across different walking conditions [37].

Comparable surface-related effects have also been reported in veterinary studies using pressure-based measurement systems. A preliminary pressure-plate study in sound ponies demonstrated that walking and trotting on a soft substrate resulted in reduced peak vertical force, vertical impulse, peak vertical pressure, and stance phase duration, accompanied by an increased hoof contact area and a more even toe–heel and mediolateral load distribution, particularly at impact. These findings support the concept that compliant surfaces dampen limb loading and promote a more homogeneous force distribution across the contact surface [38].

Veterinary research in dogs to date has focused primarily on methodological considerations. In dogs, walking on various surfaces results in significant differences in pressure-sensitive walkway measurements in both lame and non-lame individuals, including changes in PFz, and IFz. However, these studies have mainly emphasized variability in measurement outcomes depending on the surface type [39], without considering the potential therapeutic effects of walking on compliant or textured surfaces. Possible benefits related to proprioception, balance, or muscle activation remain unexplored and should be addressed in future research to evaluate the clinical relevance of surface-based interventions in veterinary rehabilitation.

The aim of this study was to evaluate the effects of surface compliance on vGRF and pCOP parameters during walk and trot in healthy adult dogs using a pressure measurement plate. For this purpose, dogs were measured at walk and trot under 4 conditions: a neutral surface and 3 yoga mats of increasing thickness. We hypothesized that pCOP parameters would significantly increase under soft-ground conditions, reflecting a greater postural stability challenge. Furthermore, we expected that the magnitude of these changes would increase with mat thickness.

## 2. Materials and Methods

### 2.1. Approval and Consent

Measurement procedure was approved by the Ethics and Animal Welfare Committee of the University of Veterinary Medicine Vienna in accordance with the guidelines for “Good Scientific Practice” (ETK-042/03/2023).

### 2.2. Animals and Inclusion Criteria

Seventeen adult client-owned dogs were enrolled in this study. Inclusion criteria comprised an age between one and ten years, a body weight between 10 and 30 kg, and no history of neurologic or orthopedic disease. All dogs underwent thorough orthopedic and neurologic examinations and were excluded if any clinical signs of musculoskeletal or neurologic abnormalities were present. A veterinarian with specialization in rehabilitation (CL) performed comprehensive orthopedic and neurologic examinations in all dogs, and excluded dogs presenting with clinical signs of musculoskeletal or neurologic abnormalities. To objectively confirm the absence of subclinical lameness or undiagnosed orthopedic conditions, only dogs with a symmetry index (SI) of PFz and IFz below 3% were included, as values above this threshold are considered indicative of lameness [12].

### 2.3. Study Protocol

#### 2.3.1. Equipment

Data were collected using a pressure measurement plate (FDM Type 2, Zebris Medical GmbH, Allgäu, Germany). The plate measures 203.2 × 54.2 cm and contains 15,360 pressure sensors, operating at a sampling frequency of 100 Hz. The measuring plate was embedded in a wooden frame to provide an even surface for the animals to step onto and was covered with a 1-mm black rubber mat. Additionally, all trials were video recorded using a Panasonic NV-MX500 camera (Panasonic, Kadoma, Osaka, Japan) to enable accurate allocation of limb contacts to the corresponding pressure data.

#### 2.3.2. Measurement Conditions

All measurements were conducted in the presence of the dogs’ owners. Before data collection, the dogs were allowed to explore the room off-leash to acclimate to the environment. Afterwards, baseline measurements as previously described [12,16,26,29,30,40] in walk and trot were performed to screen for undetected lameness and to serve as data for the neutral condition. Only dogs with a SI of PFz and IFz of less than 3% in both fore- and hindlimbs were included in the study.

Trials were conducted either off leash or on leash, with the handler consistently positioned on the same side of the dog during all on-leash trials; the handler’s side was selected based on the individual dog’s preference and was not changed between trials or experimental conditions. Each dog was walked across the pressure plate approximately 10–15 times to obtain at least 5 valid trials for both walking and trotting. A trial was considered valid if the dog moved in a straight line without changes in pace, pulling on the lead, or turning its head, and if each limb made at least 1 measurable contact with the pressure plate during that pass. Across all valid trials, a minimum of 5 stance phases per limb was required for analysis. Additionally, speed and acceleration while crossing the plate had to remain within ±0.3 m/s and ±0.5 m/s^2^, respectively [41,42,43]. To simulate different surface conditions, soft yoga mats of three thicknesses (Table 1) were placed on top of the measurement plate and the rubber mat. The measurements were performed in randomized order. For each mat condition, the same procedure as during baseline was followed until valid trials for all paws were obtained at both gaits. Between conditions, dogs were given a minimum rest period of 1 min, with additional rest provided as needed based on individual behavior and comfort. As all dogs were young and clinically healthy, no dog required extended rest periods, and none showed signs of nervousness, fatigue, or discomfort during data collection.

### 2.4. Data Analysis

Generated data were analyzed via use of the Pressure Analyzer software (Michael Schwanda, Version 4.6.5.0) and then exported to Microsoft^®^ Excel^®^ 2016. Video recordings of the measurement sessions were used to connect each limb to the correct set of data.

### 2.5. Parameters Under Investigation

The following parameters were used for evaluation:

- Mean speed (m/s) and acceleration (m/s^2^), calculated on basis of the left forelimb based on subsequent steps.
- Peak vertical force (PFz in N)
- Vertical impulse (IFz in N/s), describing the impulse in the Z-direction
- Mean duration of the stance phase (SPD) in seconds expressed as a percentage of the total stance phase duration across all limbs (SPD%):

(1) $$\mathrm{Value}\;\mathrm{in}\;\%\;\mathrm{of}\;\mathrm{total}\;\mathrm{stance}\;\mathrm{phase}\;\mathrm{duration}\;=\frac{{\mathrm{SPD}}_{\mathrm{Lx}}}{{(\mathrm{SPD}}_{\mathrm{FL}}+{\mathrm{SPD}}_{\mathrm{FR}\;}+{\mathrm{SPD}}_{\mathrm{HL}}+{\mathrm{SPD}}_{\mathrm{HR}})}*100$$

The PFz and IFz were normalized and expressed as a percentage of the total forces [16]:

(2) $$\mathrm{Value}\;\mathrm{in}\;\%\;\mathrm{of}\;\mathrm{total}\;\mathrm{force}\;=\;\frac{{XFz}_{Lx}}{{(XFz}_{FL}+{XFz}_{FR\;}+\;{XFz}_{HL}+\;{XFz}_{HR})}*100$$

where XFz is the mean value of PFz or IFz of the valid steps, Lx is the limb under investigation, FL is the left forelimb, FR is the right forelimb, HL is the let hindlimb and HR is the right hindlimb.

- Time of occurrence of PFz (TPFz) as a percentage of the stance phase of the respective limb.

Additionally, a symmetry index (SI%) was calculated as a percentage for both parameters using the following adapted equation [9,16]:

(3) $$SIXFz\;(\%)\;=abs\;(\frac{({XFz}_{LLx}-{XFz}_{RLx})\;}{\left({XFz}_{LLx}+\;{XFz}_{RLx}\right)})*100$$

Paw contact area (PCA) was defined as the effective contact surface between the paw and the pressure plate. This area was determined by summing all active sensels exceeding the pressure threshold and contributing to force transmission during stance. For each dog, PCA was calculated separately for each surface condition and gait, and these condition- and gait-specific PCA values were subsequently used for normalization.

To account for inter-individual differences in paw size and their potential influence on spatial COP parameters, all displacement-related variables were normalized to paw contact area [12,16,17,26,27]. This normalization was applied to reduce the impact of varying paw dimensions and to improve comparability between surface conditions, and gaits. By minimizing size-related variability, this approach supports robust statistical analysis and contributes to efficient study design in line with the Reduction principle of the 3R framework.

The following pCOP parameters of each paw were investigated:

COP-Area: The COP area refers to the measurement of the area covered by the COP movement. It was normalized to the paw contact area and expressed as percentage (COP-area%).

COP-Radius: The COP radius is the mean distance of all COP points to the center point of all COP points. This parameter was also normalized to the paw contact area and given as a percentage (COP-radius%).

COP-Speed is the mean velocity of the movement of the COP (COP-speed; mm/s).

The mediolateral and craniocaudal COP displacement describe the differences between the maximum positive and negative COP values on either the mediolateral or the craniocaudal axis. The mediolateral displacement was normalized to the maximum width of the paw contact area (MLD%) and the cranio-caudal displacement to the maximum length of the paw contact area (CCD%).

The craniocaudal COP displacement was calculated using the following formula:

(4) $$\mathrm{CCD}\;\left(\%\right)=\;\frac{\mathrm{COP}\;\mathrm{length}}{\mathrm{paw}\;\mathrm{length}}*100$$

The mediolateral COP displacement was calculated using this formula:

(5) $$\mathrm{MLD}\;\left(\%\right)=\frac{\mathrm{mediolateral}\;\mathrm{displacement}}{\mathrm{paw}\;\mathrm{width}}*100$$

For illustrative examples of pressure mat outputs and COP parameters in canine gait analysis, the reader is referred to Reicher et al. [12].

### 2.6. Statistical Analysis

Statistical analyses were conducted using linear mixed-effects models with restricted maximum likelihood (REML) estimation in IBM SPSS Statistics (version 29). The dependent variable was the measured outcome. Fixed effects included gait (walk, trot), surface condition (neutral, thin, medium, thick), and limb (left forelimb, right forelimb, left hindlimb, right hindlimb), as well as the three-way interactions (gait × surface condition × limb). Pairwise comparisons of estimated marginal means were performed using Sidak’s adjustment for multiple comparisons. Normality was assessed using the Shapiro–Wilk test. Statistical significance was set at *p* < 0.05.

## 3. Results

### 3.1. Animals

Fourteen dogs were included in the final analysis. The study population consisted of 9 male and 5 female dogs. The mean age was 43.21 ± 22.25 months (median: 38; range: 14–93 months), and the mean body weight was 18.97 ± 4.40 kg (median: 20.08; range: 11.00–29.40 kg).

Three dogs (2 male, 1 female) were excluded due to a SI of PFz or IFz greater than 3%, and 1 additional dog was excluded because it failed to complete 5 valid stance phases per limb on the medium-thickness mat at the trot.

### 3.2. Effects of Gait, Surface Condition, and Limb

Linear mixed-effects modeling revealed significant main effects of gait, surface condition, and limb (Supplementary Table S1).

Limb showed a significant main effect on all vGRF, indicating systematic differences between fore- and hind limbs. In addition, significant gait × limb interactions were observed.

For COP parameters, significant main effects of gait, surface condition, and limb were identified for several displacement parameters. The interactions involving surface condition × limb, gait × limb, and the three-way interaction (gait × surface condition × limb) were parameter-specific and less consistent.

Based on these overall model results, post hoc pairwise comparisons were subsequently performed to further explore condition-specific differences within gaits and limbs and are reported in the following sections.

### 3.3. Ground Reaction Forces

**Figure 1:**
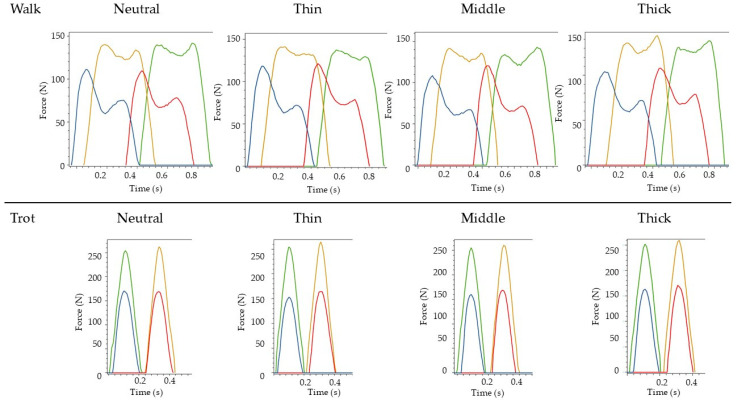
Representative vertical ground reaction force–time curves recorded with the pressure plate during walk (**top row**) and trot (**bottom row**) on the four surface conditions (Neutral, Thin, Middle, Thick). Forces are shown for the left forelimb (green), right forelimb (yellow), left hind limb (red), and right hind limb (blue). Time is displayed in seconds.

No significant differences in vGRF were observed between surface conditions during walk. During trot, almost all vGRF parameters likewise remained unchanged, except for TPFz, which was significantly increased in the right front limb when dogs trotted on the thickest mat compared to the neutral surface (Tables S2 and S3). Figure 1 shows a representative example of the vGRF–time curves recorded during the different surface conditions.

### 3.4. Paw Contact Area

**Figure 2:**
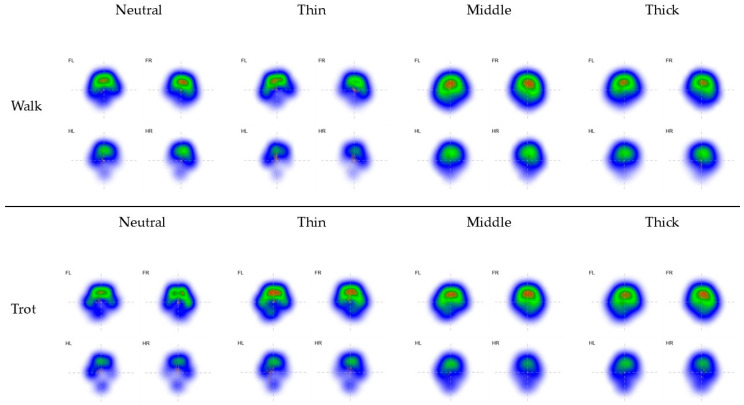
Representative pressure distribution maps illustrating changes in paw contact area (PCA) across conditions and gaits. Mean paw pressure maps are shown for walk (**top row**) and trot (**bottom row**) under neutral, thin, middle, and thick mat conditions. Forelimbs (FL, FR) and hind limbs (HL, HR) are displayed separately. With increasing mat thickness, the paw imprint becomes progressively less defined, indicating a loss of spatial detail in pressure distribution. Concurrently, the overall paw contact area increases, resulting in a broader and more diffuse pressure pattern. These visual representations illustrate condition-dependent changes in paw contact area that motivated the normalization of displacement-related parameters.

Descriptive analysis showed an overall increase in PCA with increasing mat thickness across all limbs (Table 2, Figure 2). Compared to the neutral surface PCA was significantly increased on all compliant mats except the thin mat for both walk and trot.

When comparing the compliant surfaces with each other, significant differences in PCA were observed only in the forelimbs. Specifically, PCA differed significantly between the thin and thick mats, whereas no significant differences were detected between the thin and middle mats or between the middle and thick mats.

Importantly, the pattern of significant surface-related changes in PCA was comparable between walk and trot, indicating consistent effects of surface compliance across gaits.

PCA differed significantly between experimental conditions (Table 3). With increasing mat thickness, a progressive loss of spatial detail in the paw imprint was observed, accompanied by an increase in the area covered by the paw impression (Figure 2).

### 3.5. Center of Pressure

#### 3.5.1. Walk

No significant changes were observed in COP area (%) and COP speed (mm/s). However, COP radius (%) values decreased with increasing mat thickness (Table S3). Significant changes were found in the forelimbs when comparing the neutral surface to the middle thickness and thickest mat, as well as between the thinnest and the thickest mat.

CCD% showed a significant decrease in the left front limb when comparing the neutral surface to the middle thickness and the thickest mat, as well as between the thin and thickest mat. In addition, differences were observed in the right front limb and both hind limbs when comparing the neutral surface to the thickest mat.

**Figure 3:**
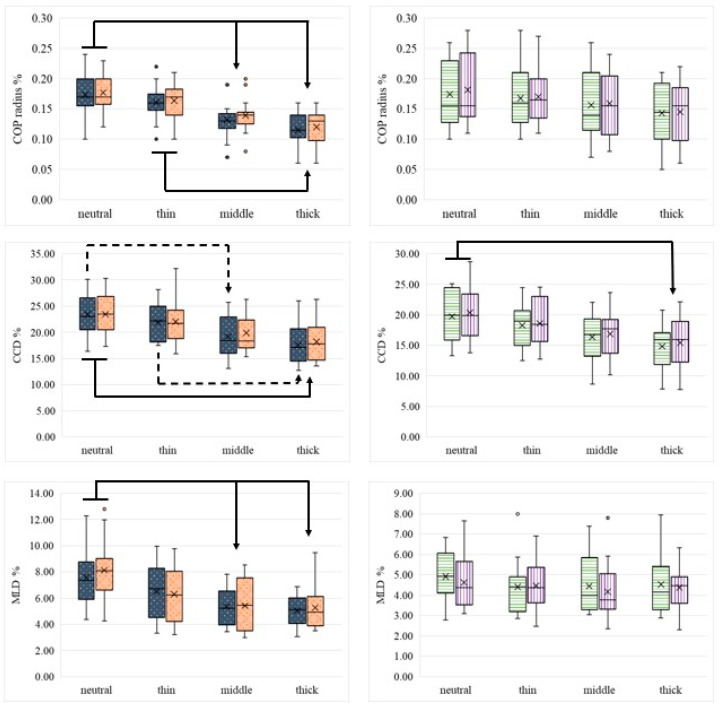
Graphical illustration of the significant differences in pCOP parameters between measurement conditions during walking for the left (grey dotted) and right (orange dotted) forelimbs, as well as the left (white and green horizontally striped) and right (white and violet vertically striped) hindlimbs. Bold arrows indicate significant differences in the limb pair, while dotted arrows indicate significant differences in only one limb. Boxplots show median values (horizontal line), interquartile range (box), and range (whiskers); crosses indicate mean values, while individual dots denote outliers.

MLD% showed a significant decrease in both front limbs when comparing the neutral surface to the middle thickness and to the thickest mat (Table 4 and Table 5, Figure 3).

Descriptive statistics and pairwise comparisons of all normalized and non-normalized parameters under investigation are provided in the Supplementary Materials (Tables S3–S5).

#### 3.5.2. Trot

In trot, the COP area (%) showed a significant decrease at the left front limb when trotting on the thickest mat compared to the thin mat. COP radius (%) decreased with increasing mat thickness. Significant differences were found in both front limbs and the right hind limb when comparing neutral trotting to the middle thickness mat. Trotting over the thickest mat resulted in a significant decrease in COP radius (%) in all limbs compared to the neutral surface. Further differences were observed in the left front limb between the thin and middle mat, and in both front limbs between the thin and thickest mat.

COP speed (mm/s) was only affected by the surface in the front limbs, showing a consistent decrease with increasing mat thickness. Comparing neutral trotting to the middle and thickest mat resulted in changes in both front limbs. Further a significant decrease was observed between the thin and thickest mat.

CCD % decreased with surface compliance and showed changes in all limbs when comparing neutral and the thin mat to both the middle and thickest mat. No significant differences in COP parameters were found when comparing trotting on the middle and thickest mat. Further, no significant differences were found in MLD % during trot (Table 6 and Table 7).

Descriptive statistics and pairwise comparisons for all normalized and non-normalized parameters are provided in the Supplementary Materials (Tables S3–S5).

## 4. Discussion

This study investigated the effects of soft ground on vGRF and pCOP parameters during walk and trot in healthy adult dogs using a pressure measurement plate. It was hypothesized that walking on softer surfaces would lead to increased pCOP displacement, reflecting a greater postural challenge. The hypothesis could not be confirmed based on the present data.

While most vGRF parameters, including PFz and IFz, remained unaffected by surface conditions, several pCOP-related variables showed consistent and significant changes. Specifically, reductions in pCOP radius, craniocaudal, and mediolateral displacement were observed with increasing mat thickness. These reductions likely reflect more controlled and constrained limb placement on compliant surfaces, indicating active neuromuscular strategies to maintain postural stability. Consistent with this interpretation, human studies have shown that walking on compliant or irregular surfaces is associated with shorter step lengths, reduced gait speed, and more compact pCOP trajectories [37,44], which are regarded as conservative adaptations to enhance stability under mechanically challenging conditions.

Importantly, the referenced human studies reporting comparable reductions in pCOP excursion employed pressure-sensing insoles, allowing for direct measurement at the foot–surface interface. While the similarity of the observed adaptations suggests comparable postural strategies in dogs, pressures in the present study were recorded beneath a compliant mat, introducing additional uncertainty. The material properties of the mat may lead to temporal delays and spatial redistribution of pressures, such that the recorded pCOP trajectories may not represent a one-to-one transfer of paw-applied forces. From a methodological perspective, surface compliance may also influence measurement sensitivity, as pressure absorption by soft mats can lead to an underestimation of vGRF and COP data [45]. Consequently, although the observed reductions in pCOP displacement are indicative of more constrained limb placement and conservative postural strategies, these interpretations should be made with appropriate caution and within the context of the chosen measurement approach.

The effects of surface compliance on pCOP parameters differed between walking and trotting, both in extent and limb involvement. In walk, vGRF and COP area (%) and COP speed (mm/s) remained unaffected by surface conditions. However, COP radius (%) decreased significantly with increasing mat thickness, particularly in the forelimbs. CCD % and MLD % also decreased, mainly in the forelimbs but also affecting both hind limbs when comparing the neutral surface to the thickest mat.

In trot, pCOP parameters were generally more sensitive to changes in surface compliance. COP radius (%) decreased significantly across all limbs with increasing mat thickness. COP area (%) showed a decrease only in the left front limb, and pCOP speed (mm/s) decreased in both front limbs with increasing surface compliance. In studies with healthy young adults, strong negative correlations between right and left foot pCOP in the mediolateral direction were observed in approximately 67% of participants, indicating consistent asymmetry during quiet standing. This pattern suggests that participants tended to shift pressure toward the lateral border of the left foot while simultaneously applying greater pressure to the medial border of the right foot [46]. Such asymmetry may reflect underlying musculoskeletal differences or leg dominance and has been discussed in the context of both functional and postural control mechanisms. Interestingly, single-leg balance tests in young adults revealed no significant differences between limbs [47,48], whereas soccer players demonstrated superior standing balance on their nondominant leg [49]. Although these findings are based on human research, a similar rationale may be applied to interpret the limb-dependent pCOP variations observed in the present study.

Unlike walking, CCD% in trot decreased consistently in all limbs when comparing the neutral and thin surfaces to the middle and thickest mats. However, in contrast to walk, no significant changes were observed in MLD% during trot, suggesting that sagittal adjustments dominated postural strategies at higher speed.

Overall, the data indicate that trot induced more uniform and global postural adaptations across all limbs, particularly in pCOP radius and CCD%, whereas adaptations observed during walk were more localized to the forelimbs and varied between parameters. Notably, mediolateral COP measures during trot did not differ from the neutral condition when dogs walked on soft surfaces, suggesting that this gait does not impose additional mediolateral postural demands.

These findings likely reflect gait-specific differences in neuromuscular control requirements. During trot, simultaneous ground contact of contralateral limb pairs provides consistent bilateral stabilization of the body, allowing the center of mass to remain close to the support line [50] and minimizing the need for additional mediolateral adjustments. Consequently, mediolateral COP control remains comparable to that observed under neutral conditions.

In contrast, during walk, postural control must be continuously adapted over the course of the stride cycle, which alternates between phases of tripedal support and bipedal support involving ipsilateral and contralateral limb pairs [50]. The absence of a consistently stabilizing stance configuration necessitates tighter mediolateral and craniocaudal COP regulation to limit body sway. Clinically sound dogs appear capable of accommodating these increased control demands through reduced COP excursions, which may explain the more variable and limb-specific changes observed in pCOP parameters during walk.

Comparable gait-dependent postural strategies have been reported in juvenile dogs, where higher pCOP values were observed during walk compared to trot, indicating that the four-beat gait imposes greater postural control demands than the two-beat gait during growth [40].

The dissociation between stable vGRF outputs and altered pCOP metrics indicates that vertical load distribution and balance control are regulated by different biomechanical systems. This finding aligns with previous studies in dogs, which demonstrated that vGRF values often remain stable even in orthopedically diseased limbs [40] or under altered limb or surface conditions, such as when wearing protective dog boots, while pCOP parameters vary significantly [26].

Our results also resonate with earlier findings in dogs walking over obstacles or performing precision tasks such as obedience heelwork, where significant pCOP adaptations occurred despite the absence of severe changes in vGRF [16,17]. A reduction in craniocaudal pCOP movement within the paws was previously connected with changes in paw pressure distribution during heel work. Similarly to this research, the findings of walking on soft surfaces can be interpreted as changes in paw roll over dynamic [16]. Together, these findings reinforce the value of pCOP metrics as sensitive indicators of subtle neuromotor adjustments. Further, it can be suggested that walking on compliant surfaces represent a challenge for proprioception and is therefore a useful tool in canine rehabilitation.

In contrast to research on dogs with osteoarthrosis, where increased pCOP excursion and area were reported due to joint instability [12], the current study found a reduction in pCOP displacement in sound dogs navigating compliant surfaces. Previous research has shown that in lame limbs, the craniocaudal pCOP displacement is shortened and cranialized compared to sound contralateral limbs [27]. This shortening of the pCOP path has been linked to a reduced swing phase and more vertical paw placement at initial contact [27,51].

In addition to mechanical joint instability, it is also possible that joint damage associated with osteoarthrosis impairs proprioceptive input from periarticular structures, thereby contributing to altered pCOP excursions. This pattern parallels the reduced pCOP displacement observed in our study on soft surfaces, suggesting that similar stabilizing strategies, such as decreased limb excursion and more vertical limb positioning, may be employed even in healthy dogs to enhance postural stability under destabilizing surface conditions.

While a reduction in pCOP displacement during gait has not been widely reported in previous canine studies, a similar pattern has been observed in static postural assessments of the body COP. In a recent study investigating the effects of visual deprivation on standing balance in dogs, adult animals exhibited a significant decrease in CCD% and COP speed when blindfolded, compared to eyes-open condition. Interestingly, blindfolding did not show a significant effect in senior dogs [30]. These findings suggest that healthy adult dogs may compensate for visual input loss by enhancing somatosensory control, particularly in the sagittal plane.

Although the context of that study was static posturography, and thus not directly comparable to dynamic gait analysis, the observed reduction in sway parameters under challenging sensory conditions aligns with the current findings. In both cases, healthy dogs appear to reduce COP displacement as part of a stabilizing strategy when confronted with instability—whether caused by surface compliance or sensory deprivation. This may reflect increased neuromuscular activity aimed at enhancing balance control, supporting the interpretation that a more compact COP path indicates an active postural stabilization mechanism.

From a methodological perspective, surface compliance can influence measurement sensitivity. Pressure absorption by soft mats may lead to an underestimation of vGRF and COP data, which has been described in related literature and must be considered when interpreting measurements obtained on compliant surfaces [45]. In the present study, PCA increased with increasing mat thickness across all limbs and both gaits. As COP displacement parameters were normalized to PCA, these condition-dependent changes in contact area may additionally influence the magnitude of normalized COP parameters. Accordingly, the observed reductions in COP excursions should be interpreted as the combined result of altered paw–surface interaction mechanics, material-dependent pressure transmission, and the applied normalization procedure.

To the best of the authors’ knowledge, no studies have specifically quantified changes in canine PCA as a function of surface compliance. A plausible explanation for the observed PCA increase is greater deformation of the compliant mats under load, leading to a redistribution of pressure over a larger contact area and a more diffuse paw imprint. As the mechanical properties of the mats were not directly quantified, this interpretation remains speculative and highlights an important area for future investigation.

This study has several limitations. Only healthy adult dogs of medium body weight were included, which limits the transferability of the results to younger, geriatric or orthopedically affected animals. Measurements were conducted under controlled laboratory conditions with straight-line gait, which may not fully reflect functional adaptations during real-world movement scenarios such as turning, accelerating, or walking on uneven natural surfaces. Furthermore, soft surfaces may have absorbed part of the applied force before it reached the sensors, potentially leading to an underestimation of vGRF and pCOP values. As a consequence, interpretations related to limb placement and postural stability are subject to additional uncertainty, as the pressure mat may not fully capture the true magnitude and spatial distribution of forces exerted at the paw–surface interface [45].

A further consideration is that limbs could have been grouped into fore- and hindlimbs to reduce model complexity. However, previous studies have shown that COP parameters can differ between left and right limbs despite symmetrical GRF [40]. Therefore, symmetry in GRF does not necessarily imply symmetry in COP behavior, and limb-specific analyses were retained to avoid masking functionally relevant effects.

Future studies should include a broader range of dog populations, including geriatric animals and those with orthopedic or neurological conditions, to evaluate whether pCOP adaptations to compliant surfaces differ with age or pathology. Combining pressure plate data with kinematic or electromyographic analysis could provide deeper insight into the neuromuscular mechanisms underlying postural adjustments. In addition, testing a wider variety of surface materials and textures—including irregular or natural substrates—would improve ecological validity and may help identify clinically relevant thresholds for instability. These approaches could support the development of standardized surface-based physiotherapeutic interventions and objective outcome measures in canine rehabilitation.

## 5. Conclusions

In summary, walking on compliant surfaces induced measurable changes in pCOP parameters but had limited effects on vGRF in healthy adult dogs. These findings suggest that dogs maintain consistent vertical loading while adjusting postural control in response to increased surface compliance. PCOP metrics—particularly COP radius and displacement—appear to be sensitive indicators of these adaptations and may prove valuable in both clinical assessment and rehabilitation planning. Future studies should combine kinetic and neuromuscular measurements and include clinical populations to better understand compensatory strategies and to refine surface-based therapeutic approaches.

## Figures and Tables

**Table 1:** Thickness of Surface Layers Used in the Experimental Setup.

	Surface Condition			
	Neutral	Thin	Middle	Thick
Thickness (mm)	1.00	5.00	8.00	10.00

**Table 2:** Mean values and standard deviations for paw contact area during walk under the following conditions: neutral (walking on a standard surface, 0.1 cm mat), thin (walking on a yoga mat with 0.5 cm thickness), middle (0.8 cm thickness), and thick (1.0 cm thickness). Measurements were obtained for the left front limb (FL), right front limb (FR), left hind limb (HL), and right hind limb (HR).

Speed	Limb	Condition			
		Neutral	Thin	Middle	Thick
Walk	FL	38.94 ± 1.50	42.77 ± 1.49	48.30 ± 1.92	49.87 ± 1.96
	FR	38.88 ± 1.39	42.70 ± 1.46	48.36 ± 1.68	50.09 ± 2.11
	HL	32.80 ± 1.60	36.94 ± 1.58	42.40 ± 1.76	42.88 ± 1.93
	HR	33.56 ± 1.55	37.05 ± 1.56	42.14 ± 1.93	43.06 ± 2.04
Trot	FL	45.73 ± 1.83	50.03 ± 1.81	56.99 ± 1.92	59.05 ± 2.27
	FR	45.39 ± 1.69	50.10 ± 1.77	57.25 ± 2.07	59.10 ± 2.24
	HL	38.57 ± 1.58	41.64 ±1.57	48.19 ± 1.79	48.68 ± 1.97
	HR	38.48 ± 1.68	41.50 ± 1.71	47.73 ± 1.83	49.21 ± 2.11

**Table 3:** *p*-values of the comparisons between conditions during walk for paw contact area for the left front limb (FL), right front limb (FR), left hind limb (HL), and right hind limb (HR). The tested conditions were as follows: neutral (walking on a standard surface, 0.1 cm mat), thin (walking on a yoga mat with 0.5 cm thickness), middle (0.8 cm thickness), and thick (1.0 cm thickness). Numbers in bold represent *p* < 0.05.

			Limb			
Speed	Condition I	Condition II	FL	FR	HL	HR
Walk	neutral	thin	0.402	0.350	0.382	0.547
		middle	**0.005**	**0.001**	**0.003**	**0.011**
		thick	**0.001**	**0.001**	**0.003**	**0.006**
	thin	neutral	0.402	0.350	0.382	0.547
		middle	0.177	0.098	0.164	0.270
		thick	**0.047**	**0.049**	0.142	0.156
	middle	neutral	**0.005**	**0.001**	**0.003**	**0.011**
		thin	0.177	0.098	0.164	0.270
		thick	0.994	0.989	1.000	1.000
	thick	neutral	**0.001**	**0.001**	**0.003**	**0.006**
		thin	**0.047**	**0.049**	0.142	0.156
		middle	0.994	0.989	1.000	1.000
Trot	neutral	thin	0.492	0.333	0.697	0.774
		middle	**0.001**	**0.001**	**0.003**	**0.006**
		thick	**0.001**	**<0.0001**	**0.003**	**0.003**
	thin	neutral	0.492	0.333	0.697	0.774
		middle	0.082	0.084	0.063	0.111
		thick	**0.028**	**0.025**	0.058	0.052
	middle	neutral	**0.001**	**0.001**	**0.003**	**0.006**
		thin	0.082	0.084	0.063	0.111
		thick	0.983	0.992	1.000	0.996
	thick	neutral	**0.001**	**<0.0001**	**0.003**	**0.003**
		thin	**0.028**	**0.025**	0.058	0.052
		middle	0.983	0.992	1.000	0.996

**Table 4:** Mean values and standard deviations for COP area (%), COP radius (%), COP speed (mm/s), craniocaudal displacement (CCD %) and mediolateral displacement (MLD %) during walk under the following conditions: neutral (walking on a standard surface, 0.1 cm mat), thin (walking on a yoga mat with 0.5 cm thickness), middle (0.8 cm thickness), and thick (1.0 cm thickness). Measurements were obtained for the left front limb (FL), right front limb (FR), left hind limb (HL), and right hind limb (HR).

		Condition			
Parameter	Limb	Neutral	Thin	Middle	Thick
COP Area (%)	FL	1.70 ± 0.21	1.42 ± 0.17	1.15 ± 0.14	1.01 ± 0.12
	FR	1.59 ± 0.19	1.39 ± 0.14	1.17 ± 0.15	1.01 ± 0.12
	HL	0.88 ± 0.10	0.75 ± 0.07	0.77 ± 0.08	0.68 ± 0.06
	HR	0.93 ± 0.09	0.82 ±0.08	0.77 ± 0.09	0.75 ± 0.08
COP Radius (%)	FL	0.17 ± 0.01	0.16 ± 0.01	0.13 ± 0.01	0.12 ± 0.01
	FR	0.18 ± 0.09	0.16 ± 0.01	0.14 ± 0.01	0.12 ± 0.01
	HL	0.17 ± 0.02	0.17 ± 0.01	0.16 ± 0.01	0.14 ± 0.01
	HR	0.18 ± 0.02	0.17 ± 0.01	0.16 ± 0.01	0.15 ± 0.01
COP Speed (mm/s)	FL	63.83 ± 2.88	60.21 ± 2.05	56.20 ± 2.03	55.63 ± 1.99
	FR	62.82 ± 2.86	60.91 ± 2.02	57.79 ± 1.82	57.15 ± 1.99
	HL	53.72 ± 3.18	53.03 ± 2.57	53.99 ± 2.59	53.59 ± 2.52
	HR	55.91 ± 3.67	54.09 ± 2.49	54.05 ± 2.26	55.20 ± 2.49
CCD (%)	FL	23.51 ± 1.06	21.90 ± 1.01	19.21 ± 1.04	17.52 ± 1.04
	FR	23.46 ± 1.04	22.02 ± 1.17	19.88 ± 0.99	18.21 ± 0.99
	HL	19.75 ± 1.11	18.29 ± 1.02	16.37 ± 1.02	14.85 ± 0.94
	HR	20.39 ± 1.19	18.60 ± 1.05	16.90 ± 1.03	15.45 ± 1.06
MLD (%)	FL	7.60 ± 0.64	6.51 ± 0.54	5.36 ± 0.42	5.05 ± 0.31
	FR	8.12 ± 0.65	6.29 ± 0.55	5.43 ± 0.54	5.27 ± 0.48
	HL	4.92 ± 0.34	4.41 ± 0.37	4.45 ± 0.37	4.54 ± 0.39
	HR	4.63 ± 0.36	4.47 ± 0.31	4.17 ± 0.38	4.37 ± 0.30

**Table 5:** *p*-values of the comparisons between conditions (CI and CII) during walk for the significantly affected COP parameters, including COP radius (%), craniocaudal displacement (CCD, %), and mediolateral displacement (MLD, %) for the left front limb (FL), right front limb (FR), left hind limb (HL), and right hind limb (HR). The tested conditions were as follows: neutral (walking on a standard surface, 0.1 cm mat), thin (walking on a yoga mat with 0.5 cm thickness), middle (0.8 cm thickness), and thick (1.0 cm thickness). Numbers in bold represent *p* < 0.05.

		COP-Radius (%)				CCD (%)				MLD (%)			
C I	C II	FL	FR	HL	HR	FL	FR	HL	HR	FL	FR	HL	HR
neutral	thin	0.924	0.851	1.000	0.995	0.864	0.935	0.92	0.849	0.754	0.222	0.899	1.000
	middle	**0.023**	**0.025**	0.952	0.887	**0.045**	0.111	0.188	0.198	**0.046**	**0.022**	0.931	0.946
	thick	**0.001**	**<0.0001**	0.566	0.446	**0.003**	**0.007**	**0.014**	**0.028**	**0.012**	**0.010**	0.976	0.996
thin	neutral	0.924	0.851	1.000	0.995	0.864	0.935	0.92	0.849	0.754	0.222	0.899	1.000
	middle	0.119	0.246	0.993	0.995	0.374	0.682	0.729	0.837	0.488	0.845	1.000	0.99
	thick	**0.003**	**0.004**	0.731	0.732	**0.033**	0.114	0.114	0.241	0.162	0.673	1.000	1.000
middle	neutral	**0.023**	**0.025**	0.952	0.887	**0.045**	0.111	0.188	0.198	**0.046**	**0.022**	0.931	0.946
	thin	0.119	0.246	0.993	0.995	0.374	0.682	0.729	0.837	0.488	0.845	1.000	0.99
	thick	0.657	0.454	0.984	0.978	0.839	0.816	0.864	0.914	0.992	1.000	1.000	0.999
thick	neutral	**0.001**	**<0.0001**	0.566	0.446	**0.003**	**0.007**	**0.014**	**0.028**	**0.012**	**0.010**	0.976	0.996
	thin	**0.003**	**0.004**	0.731	0.732	**0.033**	0.114	0.114	0.241	0.162	0.673	1.000	1.000
	middle	0.657	0.454	0.984	0.978	0.839	0.816	0.864	0.914	0.992	1.000	1.000	0.999

**Table 6:** Mean values and standard deviations for COP area (%), COP radius (%), COP speed (mm/s), craniocaudal displacement (CCD %) and mediolateral displacement (MLD %) during trot under the following conditions: neutral (standard surface, 0.1 cm mat), thin (yoga mat with 0.5 cm thickness), middle (0.8 cm thickness), and thick (1.0 cm thickness). Measurements were obtained for the left front limb (FL), right front limb (FR), left hind limb (HL), and right hind limb (HR).

		Condition			
Parameter	Limb	Neutral	Thin	Middle	Thick
COP Area (%)	FL	0.90 ± 0.16	0.82 ± 0.11	0.57 ± 0.08	0.45 ± 0.06
	FR	0.91 ± 0.13	0.80 ± 0.11	0.60 ± 0.10	0.49 ± 0.07
	HL	0.72 ± 0.09	0.65 ± 0.10	0.58 ± 0.10	0.50 ± 0.08
	HR	0.81 ± 0.14	0.69 ± 0.11	0.58 ± 0.10	0.53 ± 0.10
COP Radius (%)	FL	0.19 ± 0.01	0.17 ±0.10	0.13 ± 0.01	0.11 ± 0.01
	FR	0.19 ± 0.01	0.17 ± 0.01	0.13 ± 0.01	0.11 ± 0.01
	HL	0.19 ± 0.01	0.17 ± 0.01	0.14 ± 0.01	0.13 ± 0.01
	HR	0.19 ± 0.01	0.17 ± 0.01	0.14 ± 0.01	0.13 ± 0.01
COP speed (mm/s)	FL	143.43 ± 5.15	131.05 ± 4.48	113.54 ± 4.07	105.00 ± 4.73
	FR	145.95 ± 6.17	131.82 ± 4.73	115.86 ± 4.16	106.80 ± 4.90
	HL	137.05 ± 7.71	126.61 ± 8.27	115.27 ± 6.84	111.13 ± 8.21
	HR	142.12 ± 7.79	128.71 ± 8.72	116.07 ± 7.38	111.87 ± 8.65
CCD (%)	FL	20.09 ± 0.83	18.09 ± 0.77	13.96 ± 0.83	11.69 ± 0.76
	FR	19.88 ± 0.94	17.78 ± 0.66	14.43 ± 0.90	11.96 ± 0.79
	HL	16.28 ± 0.94	14.54 ± 0.99	12.09 ± 0.80	10.93 ± 0.81
	HR	16.65 ± 0.99	14.76 ± 0.93	12.07 ± 0.90	11.12 ± 0.89
MLD (%)	FL	3.92 ± 0.44	3.75 ± 0.44	3.48 ± 0.39	3.42 ± 0.38
	FR	4.06 ± 0.50	3.78 ± 0.39	3.21 ± 0.39	3.22 ± 0.31
	HL	3.46 ± 0.23	3.36 ± 0.19	2.91 ± 0.19	2.93 ± 0.19
	HR	3.62 ± 0.39	3.52 ± 0.33	3.06 ± 0.22	2.95 ± 0.27

**Table 7:** *p*-values of the comparisons between conditions (CI and CII) during trot for the significantly affected COP parameters, including COP radius (%), COP speed (mm/s) and craniocaudal displacement (CCD, %) for the left front limb (FL), right front limb (FR), left hind limb (HL), and right hind limb (HR). The tested conditions were as follows: neutral (walking on a standard surface, 0.1 cm mat), thin (walking on a yoga mat with 0.5 cm thickness), middle (0.8 cm thickness), and thick (1.0 cm thickness). Numbers in bold represent *p* < 0.05.

		COP Radius (%)				COP Speed (mm/s)				CCD (%)			
C I	C II	FL	FR	HL	HR	FL	FR	HL	HR	FL	FR	HL	HR
neutral	thin	0.377	0.381	0.916	0.879	0.400	0.401	0.934	0.838	0.430	0.397	0.766	0.686
	middle	**<0.0001**	**0.004**	0.078	**0.047**	**0.001**	**0.003**	0.239	0.127	**<0.0001**	**0.002**	**0.014**	**0.012**
	thick	**<0.0001**	**<0.0001**	**0.020**	**0.011**	**0.000**	**0.000**	0.165	0.088	**<0.0001**	**<0.0001**	**0.001**	**0.002**
thin	neutral	0.377	0.381	0.916	0.879	0.400	0.401	0.934	0.838	0.430	0.397	0.766	0.686
	middle	**0.029**	0.156	0.550	0.454	**0.045**	0.102	0.883	0.860	**0.007**	**0.037**	0.333	0.254
	thick	**0.000**	**0.001**	0.224	0.170	**0.003**	**0.007**	0.729	0.701	**<0.0001**	**<0.0001**	0.053	0.052
middle	neutral	**0.000**	**0.004**	0.078	**0.047**	**0.001**	**0.003**	0.239	0.127	**<0.0001**	**0.002**	**0.014**	**0.012**
	thin	**0.029**	0.156	0.550	0.454	**0.045**	0.102	0.883	0.860	**0.007**	**0.037**	0.333	0.254
	thick	0.348	0.429	0.992	0.994	0.703	0.675	0.999	0.999	0.287	0.270	0.901	0.974
thick	neutral	**<0.0001**	**<0.0001**	**0.020**	**0.011**	**<0.0001**	**<0.0001**	0.165	0.088	**<0.0001**	**<0.0001**	**0.001**	**0.002**
	thin	**<0.0001**	**0.001**	0.224	0.170	**0.003**	**0.007**	0.729	0.701	**<0.0001**	**<0.0001**	0.053	0.052
	middle	0.348	0.429	0.992	0.994	0.703	0.675	0.999	0.999	0.287	0.270	0.901	0.974

## Data Availability

The original contributions presented in this study are included in the article/Supplementary Materials. Further inquiries can be directed to the corresponding author.

## Supplementary Materials

The following supporting information can be downloaded at: https://www.mdpi.com/article/10.3390/ani16030397/s1, Table S1: Tests of fixed effects from linear mixed-effects models showing *p*-values for gait, surface condition, limb, and their interaction terms for all investigated parameters; Table S2: *p*-values of the comparisons between conditions during walk and trot were calculated for the GRF parameters, including vertical impulse (IFz %), peak vertical force (PFz %), time of occurrence of PFz (TPFz %) and stance phase duration (SPD) for the left front limb (FL), right front limb (FR), left hind limb (HL), and right hind limb (HR). The tested conditions were as follows: neutral (walking on a standard surface, 0.1 cm mat), thin (walking on a yoga mat with 0.5 cm thickness), middle (0.8 cm thickness), and thick (1.0 cm thickness); Table S3: Mean values and standard deviations for vertical Impulse (IFz), peak vertical force (PFz), stance phase duration (SPD), time of occurrence of PFz (TPFz), COP area (%), COP radius (%), COP speed (mm/s), craniocaudal displacement (CCD %) and mediolateral displacement (MLD %) during walk and trot under the following conditions: neutral (walking on a standard surface, 0.1 cm mat), thin (0.5 cm thickness), middle (0.8 cm thickness), and thick (1.0 cm thickness). Measurements were obtained for the left front limb (FL), right front limb (FR), left hind limb (HL), and right hind limb (HR); Table S4: Mean values and standard deviations of non-normalized center of pressure (COP) parameters including COP Area, COP Radius, craniocaudal displacement (CCD), mediolateral displacement (MLD) and paw contact area (PCA) during walk and trot under the following conditions: neutral (walking on a standard surface, 0.1 cm mat), thin (0.5 cm thickness), middle (0.8 cm thickness), and thick (1.0 cm thickness). Measurements were obtained for the left front limb (FL), right front limb (FR), left hind limb (HL), and right hind limb (HR); Table S5: *p*-values of the comparisons between conditions during walk and trot were calculated for the non-normalized and normalized COP parameters, including COP area, COP radius, COP speed (mm/s), craniocaudal displacement (CCD), and mediolateral displacement (MLD) for the left front limb (FL), right front limb (FR), left hind limb (HL), and right hind limb (HR). The tested conditions were as follows: neutral (walking on a standard surface, 0.1 cm mat), thin (walking on a yoga mat with 0.5 cm thickness), middle (0.8 cm thickness), and thick (1.0 cm thickness).

## Abbreviations

The following abbreviations are used in this manuscript:

CCD	Craniocaudal displacement
COP	Center of pressure
FL	Left forelimb
FR	Right forelimb
HL	Left hindlimb
HR	Right hindlimb
IFz	Vertical impulse
MLD	Mediolateral displacement
pCOP	Paw center of pressure
PFz	Peak vertical force
PS	Postural stability
SI	Symmetry index
SPD	Stance phase duration
TPFz	Time to peak vertical force
vGRF	Vertical ground reaction forces
