# Supplementary material for: Effects of Soft Ground on Paw Center of Pressure Metrics in Dogs During Walk and Trot
Source: Animals (Basel). 2026 Jan 27;16(3):397. doi: 10.3390/ani16030397 (PMC12896901; doi:10.3390/ani16030397)
Supplement: Supplementary file 1 [file animals-16-00397-s001.zip › animals-4089869-supplementary.pdf]

Supplementary Materials

Effects of Soft Ground on Paw Center of Pressure Metrics in Dogs During Walk and Trot

Christiane Lutonsky [1,*], Julia Kohlmann [1], Bianca Reicher [1], Kathleen Wittek [1], Isabella Brauner [1], Alexander Tichy [2] and Marion Mucha [1]

[1] Section of Physical Therapy and Rehabilitation, Small Animals Surgery, Clinical Centre for Small Animal Health and Research, Clinical Department for Small Animals and Horses, University of Veterinary Medicine, 1210 Vienna, Austria.
[2] Department of Biological Sciences and Pathobiology, Platform for Bioinformatics and Biostatistics, Centre of Biological Sciences, University of Veterinary Medicine, 1210 Vienna, Austria
* Correspondence: christiane.lutonsky@vetmeduni.ac.at

Table S1. Tests of fixed effects from linear mixed-effects models showing p-values for gait, surface condition, limb, and their interaction terms for all investigated parameters.

Source	Parameter									
	PFz	IFz	TPFz	SPD	PCA	COP Area (%)	COP Radius (%)	COP Speed (mm/s)	CLD (%)	MLD (%)
**Gait**	0,999	0,999	<0.0001	0,999	<0.0001	<0.0001	0,705	<0.0001	<0.0001	<0.0001
**Condition**	1,000	1,000	0,074	1,000	<0.0001	<0.0001	<0.0001	<0.0001	<0.0001	<0.0001
**Limb**	<0.0001	<0.0001	<0.0001	<0.0001	<0.0001	<0.0001	0,100	0,339	<0.0001	<0.0001
**Gait * Condition**	1,000	1,000	0,300	1,000	0,746	0,932	0,101	<0.0001	0,298	0,147
**Gait * Limb**	<0.0001	<0.0001	<0.0001	<0.0001	0,258	<0.0001	0,657	0,617	0,725	<0.0001

**Condition * Limb**	0,558	0,863	0,722	0,940	0,996	0,278	0,762	0,908	0,835	0,298
**Gait * Condition * Limb**	0,960	0,477	0,979	0,025	1,000	0,997	1,000	1,000	0,998	0,348

Table S2. P-values of the comparisons between conditions during walk and trot were calculated for the GRF parameters, including vertical impulse (IFz %), peak vertical force (PFz %), time of occurrence of PFz (TPFz %) and stance phase duration (SPD) for the left front limb (FL), right front limb (FR), left hind limb (HL), and right hind limb (HR). The tested conditions were as follows: neutral (walking on a standard surface, 0.1 cm mat), thin (walking on a yoga mat with 0.5 cm thickness), middle (0.8 cm thickness), and thick (1.0 cm thickness).

**Speed**	**Limb**	**Condition I**	**Condition II**	**IFz (%)**	**TPFz**	**PFz (%)**	**SPD**
			**thin**	0,994	1,000	0,898	0,983
		**neutral**	**middle**	0,960	0,823	0,907	0,888
			**thick**	0,957	0,977	0,857	0,963
			**neutral**	0,994	1,000	0,898	0,983
		**thin**	**middle**	1,000	0,675	1,000	0,997
			**thick**	1,000	0,918	1,000	1,000
	FL		**neutral**	0,960	0,823	0,907	0,888
		**middle**	**thin**	1,000	0,675	1,000	0,997
			**thick**	1,000	0,996	1,000	1,000
Walk			**neutral**	0,957	0,977	0,857	0,963
		**thick**	**thin**	1,000	0,918	1,000	1,000
			**middle**	1,000	0,996	1,000	1,000
			**thin**	0,484	0,999	0,947	0,703
		**neutral**	**middle**	0,994	0,939	0,998	0,607
			**thick**	0,994	0,994	0,972	0,967
	FR		**neutral**	0,484	0,999	0,947	0,703
		**thin**	**middle**	0,933	0,795	0,998	1,000
			**thick**	0,946	0,939	1,000	0,999
		**middle**	**neutral**	0,994	0,939	0,998	0,607

		**thin**	0,933	0,795	0,998	1,000
		**thick**	1,000	0,999	1,000	1,000
		**neutral**	0,994	0,994	0,972	0,967
	**thick**	**thin**	0,946	0,939	1,000	0,999
		**middle**	1,000	0,999	1,000	1,000
		**thin**	0,875	0,820	0,955	0,862
	**neutral**	**middle**	0,900	0,961	0,904	0,765
		**thick**	0,977	1,000	0,895	0,996
		**neutral**	0,875	0,820	0,955	0,862
	**thin**	**middle**	1,000	0,997	1,000	1,000
		**thick**	0,999	0,808	1,000	0,998
HL		**neutral**	0,900	0,961	0,904	0,765
	**middle**	**thin**	1,000	0,997	1,000	1,000
		**thick**	1,000	0,956	1,000	0,992
		**neutral**	0,977	1,000	0,895	0,996
	**thick**	**thin**	0,999	0,808	1,000	0,998
		**middle**	1,000	0,956	1,000	0,992
		**thin**	0,701	0,956	0,895	0,959
	**neutral**	**middle**	0,986	1,000	0,999	0,870
		**thick**	0,899	1,000	0,956	0,855
		**neutral**	0,701	0,956	0,895	0,959
	**thin**	**middle**	0,981	0,992	0,982	1,000
		**thick**	0,999	0,969	1,000	1,000
HR		**neutral**	0,986	1,000	0,999	0,870
	**middle**	**thin**	0,981	0,992	0,982	1,000
		**thick**	1,000	1,000	0,997	1,000
		**neutral**	0,899	1,000	0,956	0,855
	**thick**	**thin**	0,999	0,969	1,000	1,000
		**middle**	1,000	1,000	0,997	1,000

Table S3. Mean values and standard deviations for vertical Impulse (IFz), peak vertical force (PFz), stance phase duration (SPD), time of occurrence of PFz (TPFz), COP area (%), COP radius (%), COP speed (mm/s), craniocaudal displacement (CCD %) and mediolateral displacement (MLD %) during walk and trot under the following conditions: neutral (walking on a standard surface, 0.1 cm mat), thin (0.5 cm thickness), middle (0.8 cm thickness), and thick (1.0 cm thickness). Measurements were obtained for the left front limb (FL), right front limb (FR), left hind limb (HL), and right hind limb (HR).

Speed	Parameter	Limb	Neutral	Thin	Middle	Thick
Walk	IFz (%)	FL	31,77 ± 0,25	31,57 ± 0,26	31,49 ± 0,23	31,49 ± 0,22
		FR	31,42 ± 0,21	30,91 ± 0,22	31,23 ± 0,26	31,22 ± 0,27
		HL	18,31 ± 0,21	18,64 ± 0,22	18,61 ± 0,21	18,52 ± 0,19
		HR	18,5 ± 0,18	18,88 ± 0,22	18,67 ± 0,20	18,77 ± 0,21
	PFz (%)	FL	29,64 ± 0,42	29,10 ± 0,32	29,12 ± 0,30	29,04 ± 0,34
		FR	29,43 ± 0,37	28,96 ± 0,38	29,20 ± 0,34	29,03 ± 0,34
		HL	20,46 ± 0,41	20,93 ± 0,37	20,98 ± 0,32	21,00 ± 0,33
		HR	20,48 ± 0,41	21,01 ± 0,33	20,70 ± 0,30	20,93 ± 0,35
	SPD (%)	FL	26,08 ± 0,22	25,90 ± 0,13	25,81 ± 0,14	25,87 ± 0,14
		FR	25,89 ± 0,10	25,65 ± 0,14	25,68 ± 0,10	25,74 ± 0,16
		HL	23,92 ± 0,17	24,16 ± 0,15	24,19 ± 0,13	24,05 ± 0,18
		HR	24,12 ± 0,16	24,29 ± 0,13	24,33 ± 0,12	24,35 ± 0,13
	TPFz (%)	FL	40,48 ± 4,33	39,66 ± 3,56	48,03 ± 4,75	44,83 ± 3,96
		FR	39,90 ± 3,58	37,89 ± 3,76	45,31 ± 4,76	43,00 ± 4,18
		HL	24,47 ± 1,72	22,09 ± 1,03	22,83 ± 0,99	24,51 ± 1,72
		HR	23,62 ± 1,37	22,19 ± 0,99	23,02 ± 0,97	23,68 ± 1,61
	COP Area (%)	FL	1,70 ± 0,21	1,42 ± 0,17	1,15 ± 0,14	1,01 ± 0,12
		FR	1,59 ± 0,19	1,39 ± 0,14	1,17 ± 0,15	1,01 ± 0,12
		HL	0,88 ± 0,10	0,75 ± 0,07	0,77 ± 0,08	0,68 ± 0,06
		HR	0,93 ± 0,09	0,82 ±0,08	0,77 ± 0,09	0,75 ± 0,08
	COP Radius (%)	FL	0,17 ± 0,01	0,16 ± 0,01	0,13 ± 0,01	0,12 ± 0,01
		FR	0,18 ± ,009	0,16 ± 0,01	0,14 ± 0,01	0,12 ± 0,01
		HL	0,17 ± 0,02	0,17 ± 0,01	0,16 ± 0,01	0,14 ± 0,01
		HR	0,18 ± 0,02	0,17 ± 0,01	0,16 ± 0,01	0,15 ± 0,01
	COP speed (mm/s)	FL	63,83 ± 2,88	60,21 ± 2,05	56,20 ± 2,03	55,63 ± 1,99
		FR	62,82 ± 2,86	60,91 ± 2,02	57,79 ± 1,82	57,15 ± 1,99

		HL	53,72 ± 3,18	53,03 ± 2,57	53,99 ± 2,59	53,59 ± 2,52
		HR	55,91 ± 3,67	54,09 ± 2,49	54,05 ± 2,26	55,20 ± 2,49
	CCD (%)	FL	23,51 ± 1,06	21,90 ± 1,01	19,21 ± 1,04	17,52 ± 1,04
		FR	23,46 ± 1,04	22,02 ± 1,17	19,88 ± 0,99	18,21 ± 0,99
		HL	19,75 ± 1,11	18,29 ± 1,02	16,37 ± 1,02	14,85 ± 0,94
		HR	20,39 ± 1,19	18,60 ± 1,05	16,90 ± 1,03	15,45 ± 1,06
	MLD (%)	FL	7,60 ± 0,64	6,51 ± 0,54	5,36 ± 0,42	5,05 ± 0,31
		FR	8,12 ± 0,65	6,29 ± 0,55	5,43 ± 0,54	5,27 ± 0,48
		HL	4,92 ± 0,34	4,41 ± 0,37	4,45 ± 0,37	4,54 ± 0,39
		HR	4,63 ± 0,36	4,47 ± 0,31	4,17 ± 0,38	4,37 ± 0,30
	IFz (%)	FL	31,94 ± 0,23	31,80 ± 0,21	31,63 ± 0,29	31,66 ± 0,23
		FR	31,43 ± 0,20	31,81 ± 0,18	31,78 ± 0,19	31,57 ± 0,23
		HL	18,38 ± 0,19	18,25 ± 0,20	18,29 ± 0,21	18,23 ± 0,20
		HR	18,26 ± 0,19	18,17 ± 0,15	18,30 ± 0,26	18,53 ± 0,23
	PFz (%)	FL	30,11 ± 0,19	29,92 ± 0,16	29,68 ± 0,23	29,80 ± 0,17
		FR	29,97 ± 0,26	30,01 ± 0,23	29,84 ± 0,26	29,79 ± 0,21
		HL	20,05 ± 0,19	20,11 ± 0,21	20,29 ± 0,18	20,20 ±0,10
		HR	19,87 ± 0,19	19,96 ± 0,15	20,19 ± 0,24	20,22 ± 0,19
	SPD (%)	FL	27,03 ± 0,17	27,31 ± 0,15	27,23 ± 0,16	27,14 ± 0,15
		FR	26,92 ± 0,14	27,36 ± 0,15	27,33 ± 0,11	27,24 ± 0,17
		HL	23,08 ± 0,18	22,73 ± 0,18	22,80 ± 0,22	22,75 ± 0,14
Trot		HR	22,96 ± 0,11	22,60 ± 0,14	22,66 ± 0,14	22,88 ± 0,16
	TPFz (%)	FL	41,48 ± 0,72	42,43 ± 0,60	43,80 ± 0,79	43,98 ± 0,60
		FR	41,20 ± 0,63	42,86 ± 0,67	43,43 ± 0,59	44,09 ± 0,54
		HL	41,15 ± 0,57	41,63 ± 0,43	41,01 ± 0,46	41,45 ± 0,46
		HR	40,71 ± 0,53	40,96 ± 0,27	41,32 ± 0,36	41,23 ± 0,37
	COP Area (%)	FL	0,90 ± 0,16	0,82 ± 0,11	0,57 ± 0,08	0,45 ± 0,06
		FR	0,91 ± 0,13	0,80 ± 0,11	0,60 ± 0,10	0,49 ± 0,07
		HL	0,72 ± 0,09	0,65 ± 0,10	0,58 ± 0,10	0,50 ± 0,08
		HR	0,81 ± 0,14	0,69 ± 0,11	0,58 ± 0,10	0,53 ± 0,10
	COP Radius (%)	FL	0,19 ± 0,01	0,17 ±0,10	0,13 ± 0,01	0,11 ± 0,01
		FR	0,19 ± 0,01	0,17 ± 0,01	0,13 ± 0,01	0,11 ± 0,01
		HL	0,19 ± 0,01	0,17 ± 0,01	0,14 ± 0,01	0,13 ± 0,01

	HR	0,19 ± 0,01	0,17 ± 0,01	0,14 ± 0,01	0,13 ± 0,01
COP speed (mm/s)	FL	143,43 ± 5,15	131,05 ± 4,48	113,54 ± 4,07	105,00 ± 4,73
	FR	145,95 ± 6,17	131,82 ± 4,73	115,86 ± 4,16	106,80 ± 4,90
	HL	137,05 ± 7,71	126,61 ± 8,27	115,27 ± 6,84	111,13 ± 8,21
	HR	142,12 ± 7,79	128,71 ± 8,72	116,07 ± 7,38	111,87 ± 8,65
CCD (%)	FL	20,09 ± 0,83	18,09 ± 0,77	13,96 ± 0,83	11,69 ± 0,76
	FR	19,88 ± 0,94	17,78 ± 0,66	14,43 ± 0,90	11,96 ± 0,79
	HL	16,28 ± 0,94	14,54 ± 0,99	12,09 ± 0,80	10,93 ± 0,81
	HR	16,65 ± 0,99	14,76 ± 0,93	12,07 ± 0,90	11,12 ± 0,89
MLD (%)	FL	3,92 ± 0,44	3,75 ± 0,44	3,48 ± 0,39	3,42 ± 0,38
	FR	4,06 ± 0,50	3,78 ± 0,39	3,21 ± 0,39	3,22 ± 0,31
	HL	3,46 ± 0,23	3,36 ± 0,19	2,91 ± 0,19	2,93 ± 0,19
	HR	3,62 ± 0,39	3,52 ± 0,33	3,06 ± 0,22	2,95 ± 0,27

Table S4. Mean values and standard deviations of non-normalized center of pressure (COP) parameters including COP Area, COP Radius, craniocaudal displacement (CCD), mediolateral displacement (MLD) and paw contact area (PCA) during walk and trot under the following conditions: neutral (walking on a standard surface, 0.1 cm mat), thin (0.5 cm thickness), middle (0.8 cm thickness), and thick (1.0 cm thickness). Measurements were obtained for the left front limb (FL), right front limb (FR), left hind limb (HL), and right hind limb (HR).

**Speed**	**Parameter**	**Limb**	**Condition**			
			Neutral	Thin	Middle	Thick
Walk	COP Area (mm2)	FL	45.85 ± 5.59	36.55 ± 4.43	30.96 ± 3.98	26.15 ± 3.01
		FR	42.75 ± 5.05	36.49 ± 3.32	31.55 ± 3.98	27.35 ± 3.25
		HL	20.11 ± 2.51	17.73 ± 1.85	18.08 ± 1.84	16.12 ± 1.64
		HR	21.81 ± 2.30	19.50 ± 1.85	17.94 ± 1.93	17.43 ± 1.87
	COP Radius (mm)	FL	4.69 ± 0.26	4.39 ± 0.22	3.60 ± 0.24	3.25 ± 0.26
		FR	4.74 ± 0.22	4.41 ± 0.23	3.78 ± 0.22	3.34 ± 0.23
		HL	4.00 ± 0.38	3,88 ± 0.34	3.75 ± 0.35	3.32 ± 0.32
		HR	4.23 ± 0.40	3,95 ± 0.33	3.71 ± 0.35	3.39 ± 0.34

	CCD (mm)	FL	18.76 ± 1.15	18.33 ± 1.15	16.71 ± 1.25	15.63 ± 1.15
		FR	18.76 ± 1.03	18.09 ± 1.10	17.30 ± 1.08	16.05 ± 1.10
		HL	15.04 ± 1.22	14.70 ± 1.12	14.02 ± 1.21	12.72 ± 1.01
		HR	15.99 ± 1.29	15.01 ± 1.11	14.22 ± 1.15	13.30 ± 1.14
	MLD (mm)	FL	5.31 ± 0.49	4,45 ± 0.44	4.10 ± 0.34	3.70 ± 0.28
		FR	5.62 ± 0.49	4.29 ± 0.39	4.23 ± 0.48	3.86 ± 0.36
		HL	2.93 ± 0.23	2.78 ± 0.26	3.04 ± 0.28	3.08 ± 0.29
		HR	2.80 ± 0.25	2.87 ± 0.25	2.81 ± 0.27	3.04 ± 0.24
	PCA (mm2)	FL	38.94 ± 1.50	42.77 ± 1.49	48.30 ± 1.92	49.87 ± 1.96
		FR	38.88 ± 1.39	42.70 ± 1.46	48.36 ± 1.68	50.09 ± 2.11
		HL	32.80 ± 1.60	36.94 ± 1.58	42.40 ± 1.76	42.88 ± 1.93
		HR	33.56 ± 1.55	37.05 ± 1.56	42.14 ± 1.93	43.06 ± 2.04
	COP Area (mm2)	FL	25.48 ± 4.37	21.80 ± 2.91	18.02 ± 3.20	12.41 ± 1.72
		FR	24.65 ± 3.60	20.91 ± 2.55	18.17 ± 4.05	13.25 ± 1.83
		HL	16.35 ± 1.98	14.65 ± 2.00	12.71 ± 1.96	11.28 ± 1.83
		HR	18.61 ± 3.29	16.06 ± 2.70	13.41 ± 2.58	12.18 ± 2.38
	COP Radius (mm)	FL	5.08 ± 0.30	4.53 ± 0.24	3.58 ± 0.24	3.00 ± 0.19
		FR	5.19 ± 0.31	4.49 ± 0.25	3.68 ± 0.28	3.03 ± 0.21
		HL	4.21 ± 0.30	3.76 ± 0.30	3.24 ± 0.28	2.93 ± 0.26
		HR	4.37 ± 0.34	3.81 ± 0.32	3.27 ± 0.32	2.94 ± 0.27
Trot	CCD (mm)	FL	17.39 ± 0.93	15.88 ± 0.85	13.38 ± 1.08	11.39 ± 0.92
		FR	17.79 ± 0.98	15.96 ± 0.89	14.00 ± 1.19	11.70 ± 0.91
		HL	13.97 ± 1.07	12,53 ± 1.04	11.20 ± 1.07	10.09 ± 0.91
		HR	14.56 ± 1.11	12.76 ± 1.10	11.23 ± 1.13	10.33 ± 0.99
	MLD (mm)	FL	3.16 ± 0.39	2.92 ± 0.32	3.04 ± 0.36	2.80 ± 0.28
		FR	3.20 ± 0.44	2.91 ± 0.28	2.89 ± 0.45	2.63 ± 0.24
		HL	2.36 ± 0.18	2.29 ± 0.15	2.10 ± 0.15	2.12 ± 0.16
		HR	2.46 ± 0.24	2.37 ± 0.21	2.29 ± 0.22	2.18 ± 0.24
	PCA (mm2)	FL	45.73 ± 1.83	50.03 ± 1.81	56.99 ± 1.92	59.05 ± 2.27
		FR	45.39 ± 1.69	50.10 ± 1.77	57.25 ± 2.07	59.10 ± 2.24
		HL	38.57 ± 1.58	41.64 ±1.57	48.19 ± 1.79	48.68 ± 1.97
		HR	38.48 ± 1.68	41.50 ± 1.71	47.73 ± 1.83	49.21 ± 2.11

Table S5. P-values of the comparisons between conditions during walk and trot were calculated for the non-normalized and normalized COP parameters, including COP area, COP radius, COP speed (mm/s), craniocaudal displacement (CCD), and mediolateral displacement (MLD) for the left front limb (FL), right front limb (FR), left hind limb (HL), and right hind limb (HR). The tested conditions were as follows: neutral (walking on a standard surface, 0.1 cm mat), thin (walking on a yoga mat with 0.5 cm thickness), middle (0.8 cm thickness), and thick (1.0 cm thickness).

				Parameter									
**Speed**	**Limb**	**Condition I**	**Condition II**	**COP Area (mm2)**	**COP Area (%)**	**COP Radius (mm)**	**COP Radius (%)**	**COP Speed (mm/s)**	**CCD (mm)**	**CCD (%)**	**MLD (mm)**	**MLD (%)**	**PCA**
Walk	FL	**neutral**	**thin**	0,746	0,889	0,954	0,924	0,897	1,000	0,864	0,742	0,754	0,402
			**middle**	0,219	0,213	0,028	0,023	0,222	0,802	0,045	0,289	0,046	0,005
			**thick**	0,033	0,051	0,004	0,001	0,157	0,334	0,003	0,056	0,012	0,001
		**thin**	**neutral**	0,746	0,889	0,954	0,924	0,897	1,000	0,864	0,742	0,754	0,402
			**middle**	0,929	0,785	0,121	0,119	0,688	0,923	0,374	0,991	0,488	0,177
			**thick**	0,330	0,288	0,015	0,003	0,538	0,499	0,033	0,655	0,162	0,047
		**middle**	**neutral**	0,219	0,213	0,028	0,023	0,222	0,802	0,045	0,289	0,046	0,005
			**thin**	0,929	0,785	0,121	0,119	0,688	0,923	0,374	0,991	0,488	0,177
			**thick**	0,920	0,976	0,912	0,657	1,000	0,989	0,839	0,932	0,992	0,994
		**thick**	**neutral**	0,033	0,051	0,004	0,001	0,157	0,334	0,003	0,056	0,012	0,001
			**thin**	0,330	0,288	0,015	0,003	0,538	0,499	0,033	0,655	0,162	0,047
			**middle**	0,920	0,976	0,912	0,657	1,000	0,989	0,839	0,932	0,992	0,994
	FR	**neutral**	**thin**	0,894	0,963	0,891	0,851	0,995	0,998	0,935	0,236	0,222	0,350
			**middle**	0,448	0,434	0,027	0,025	0,627	0,915	0,111	0,272	0,022	0,001
			**thick**	0,102	0,103	0,001	0,000	0,526	0,412	0,007	0,046	0,010	0,001
		**thin**	**neutral**	0,894	0,963	0,891	0,851	0,995	0,998	0,935	0,236	0,222	0,350
			**middle**	0,925	0,856	0,288	0,246	0,837	0,997	0,682	1,000	0,845	0,098
			**thick**	0,311	0,280	0,017	0,004	0,730	0,742	0,114	0,966	0,673	0,049
		**middle**	**neutral**	0,448	0,434	0,027	0,025	0,627	0,915	0,111	0,272	0,022	0,001
			**thin**	0,925	0,856	0,288	0,246	0,837	0,997	0,682	1,000	0,845	0,098
			**thick**	0,963	0,964	0,689	0,454	1,000	0,965	0,816	0,991	1,000	0,989
		**thick**	**neutral**	0,102	0,103	0,001	0,000	0,526	0,412	0,007	0,046	0,010	0,001
			**thin**	0,311	0,280	0,017	0,004	0,730	0,742	0,114	0,966	0,673	0,049

			**middle**	0,963	0,964	0,689	0,454	1,000	0,965	0,816	0,991	1,000	0,989
			**thin**	0,973	0,904	1,000	1,000	1,000	1,000	0,920	0,998	0,899	0,382
		**neutral**	**middle**	0,988	0,957	0,997	0,952	1,000	0,992	0,188	1,000	0,931	0,003
			**thick**	0,730	0,522	0,698	0,566	1,000	0,638	0,014	0,999	0,976	0,003
			**neutral**	0,973	0,904	1,000	1,000	1,000	1,000	0,920	0,998	0,899	0,382
		**thin**	**middle**	1,000	1,000	1,000	0,993	1,000	0,999	0,729	0,982	1,000	0,164
	HL		**thick**	0,988	0,973	0,807	0,731	1,000	0,742	0,114	0,966	1,000	0,142
			**neutral**	0,988	0,957	0,997	0,952	1,000	0,992	0,188	1,000	0,931	0,003
		**middle**	**thin**	1,000	1,000	1,000	0,993	1,000	0,999	0,729	0,982	1,000	0,164
			**thick**	0,966	0,937	0,943	0,984	1,000	0,961	0,864	1,000	1,000	1,000
			**neutral**	0,730	0,522	0,698	0,566	1,000	0,638	0,014	0,999	0,976	0,003
		**thick**	**thin**	0,988	0,973	0,807	0,731	1,000	0,742	0,114	0,966	1,000	0,142
			**middle**	0,966	0,937	0,943	0,984	1,000	0,961	0,864	1,000	1,000	1,000
			**thin**	0,977	0,942	0,996	0,995	0,999	0,993	0,849	1,000	1,000	0,547
		**neutral**	**middle**	0,755	0,730	0,914	0,887	0,999	0,897	0,198	1,000	0,946	0,011
			**thick**	0,628	0,615	0,537	0,446	1,000	0,565	0,028	0,984	0,996	0,006
			**neutral**	0,977	0,942	0,996	0,995	0,999	0,993	0,849	1,000	1,000	0,547
		**thin**	**middle**	0,995	0,998	0,997	0,995	1,000	0,997	0,837	1,000	0,990	0,270
	HR		**thick**	0,977	0,989	0,818	0,732	1,000	0,874	0,241	0,998	1,000	0,156
			**neutral**	0,755	0,730	0,914	0,887	0,999	0,897	0,198	1,000	0,946	0,011
		**middle**	**thin**	0,995	0,998	0,997	0,995	1,000	0,997	0,837	1,000	0,990	0,270
			**thick**	1,000	1,000	0,986	0,978	1,000	0,994	0,914	0,990	0,999	1,000
			**neutral**	0,628	0,615	0,537	0,446	1,000	0,565	0,028	0,984	0,996	0,006
		**thick**	**thin**	0,977	0,989	0,818	0,732	1,000	0,874	0,241	0,998	1,000	0,156
			**middle**	1,000	1,000	0,986	0,978	1,000	0,994	0,914	0,990	0,999	1,000
			**thin**	0,983	0,999	0,656	0,377	0,400	0,809	0,430	0,998	1,000	0,492
		**neutral**	**middle**	0,699	0,370	0,004	0,000	0,001	0,055	0,000	1,000	0,975	0,001
			**thick**	0,074	0,094	0,000	0,000	0,000	0,001	0,000	0,979	0,951	0,001
Trot	FL		**neutral**	0,983	0,999	0,656	0,377	0,400	0,809	0,430	0,998	1,000	0,492
		**thin**	**middle**	0,948	0,368	0,056	0,029	0,045	0,398	0,007	1,000	0,998	0,082
			**thick**	0,065	0,045	0,000	0,000	0,003	0,008	0,000	1,000	0,994	0,028
		**middle**	**neutral**	0,699	0,370	0,004	0,000	0,001	0,055	0,000	1,000	0,975	0,001
			**thin**	0,948	0,368	0,056	0,029	0,045	0,398	0,007	1,000	0,998	0,082

		**thick**	0,590	0,807	0,348	0,348	0,703	0,680	0,287	0,997	1,000	0,983
		**neutral**	0,074	0,094	0,000	0,000	0,000	0,001	0,000	0,979	0,951	0,001
	**thick**	**thin**	0,065	0,045	0,000	0,000	0,003	0,008	0,000	1,000	0,994	0,028
		**middle**	0,590	0,807	0,348	0,348	0,703	0,680	0,287	0,997	1,000	0,983
		**thin**	0,956	0,992	0,435	0,381	0,401	0,694	0,397	0,995	0,999	0,333
	**neutral**	**middle**	0,812	0,377	0,007	0,004	0,003	0,121	0,002	0,997	0,729	0,001
		**thick**	0,063	0,062	0,000	0,000	0,000	0,001	0,000	0,841	0,679	0,000
		**neutral**	0,956	0,992	0,435	0,381	0,401	0,694	0,397	0,995	0,999	0,333
	**thin**	**middle**	0,994	0,722	0,221	0,156	0,102	0,736	0,037	1,000	0,888	0,084
FR		**thick**	0,129	0,148	0,001	0,001	0,007	0,015	0,000	0,974	0,848	0,025
		**neutral**	0,812	0,377	0,007	0,004	0,003	0,121	0,002	0,997	0,729	0,001
	**middle**	**thin**	0,994	0,722	0,221	0,156	0,102	0,736	0,037	1,000	0,888	0,084
		**thick**	0,865	0,921	0,365	0,429	0,675	0,593	0,270	0,996	1,000	0,992
		**neutral**	0,063	0,062	0,000	0,000	0,000	0,001	0,000	0,841	0,679	0,000
	**thick**	**thin**	0,129	0,148	0,001	0,001	0,007	0,015	0,000	0,974	0,848	0,025
		**middle**	0,865	0,921	0,365	0,429	0,675	0,593	0,270	0,996	1,000	0,992
		**thin**	0,992	0,996	0,889	0,916	0,934	0,921	0,766	1,000	1,000	0,697
	**neutral**	**middle**	0,742	0,882	0,154	0,078	0,239	0,385	0,014	0,843	0,398	0,003
		**thick**	0,359	0,372	0,021	0,020	0,165	0,063	0,001	0,903	0,423	0,003
		**neutral**	0,992	0,996	0,889	0,916	0,934	0,921	0,766	1,000	1,000	0,697
	**thin**	**middle**	0,983	0,995	0,775	0,550	0,883	0,942	0,333	0,940	0,503	0,063
HL		**thick**	0,784	0,766	0,250	0,224	0,729	0,433	0,053	0,972	0,534	0,058
		**neutral**	0,742	0,882	0,154	0,078	0,239	0,385	0,014	0,843	0,398	0,003
	**middle**	**thin**	0,983	0,995	0,775	0,550	0,883	0,942	0,333	0,940	0,503	0,063
		**thick**	0,996	0,990	0,962	0,992	0,999	0,969	0,901	1,000	1,000	1,000
		**neutral**	0,359	0,372	0,021	0,020	0,165	0,063	0,001	0,903	0,423	0,003
	**thick**	**thin**	0,784	0,766	0,250	0,224	0,729	0,433	0,053	0,972	0,534	0,058
		**middle**	0,996	0,990	0,962	0,992	0,999	0,969	0,901	1,000	1,000	1,000
		**thin**	0,992	0,985	0,802	0,879	0,838	0,834	0,686	1,000	1,000	0,774
	**neutral**	**middle**	0,783	0,709	0,145	0,047	0,127	0,244	0,012	0,996	0,785	0,006
HR		**thick**	0,554	0,520	0,016	0,011	0,088	0,050	0,002	0,958	0,676	0,003
	**thin**	**neutral**	0,992	0,985	0,802	0,879	0,838	0,834	0,686	1,000	1,000	0,774
		**middle**	0,981	0,971	0,814	0,454	0,860	0,919	0,254	1,000	0,833	0,111

		**thick**	0,872	0,871	0,252	0,170	0,701	0,510	0,052	0,993	0,722	0,052
		**neutral**	0,783	0,709	0,145	0,047	0,127	0,244	0,012	0,996	0,785	0,006
	**middle**	**thin**	0,981	0,971	0,814	0,454	0,860	0,919	0,254	1,000	0,833	0,111
		**thick**	1,000	1,000	A0,969	0,994	0,999	0,992	0,974	1,000	1,000	0,996
		**neutral**	0,554	0,520	0,016	0,011	0,088	0,050	0,002	0,958	0,676	0,003
	**thick**	**thin**	0,872	0,871	0,252	0,170	0,701	0,510	0,052	0,993	0,722	0,052
		**middle**	1,000	1,000	0,969	0,994	0,999	0,992	0,974	1,000	1,000	0,996
